# Mapping EQ-5D-3L from the Knee Injury and Osteoarthritis Outcome Score (KOOS)

**DOI:** 10.1007/s11136-019-02303-9

**Published:** 2019-09-20

**Authors:** Ali Kiadaliri, Monica Hernández Alava, Ewa M. Roos, Martin Englund

**Affiliations:** 1 Clinical Epidemiology Unit, Department of Clinical Sciences Lund, Orthopaedics, Faculty of Medicine, Lund University, Remissgatan 4, 221 85 Lund, Sweden; 2 Centre for Economic Demography, Lund University, Lund, Sweden; 3 Health Economics and Decision Science, University of Sheffield, Sheffield, UK; 4 Department of Sports Science and Clinical Biomechanics, University of Southern Denmark, Odense, Denmark; 5 Clinical Epidemiology Research and Training Unit, Boston University School of Medicine, Boston, MA USA

**Keywords:** EQ-5D-3L, KOOS, Mapping, Beta-mixture, Linear regression, Response mapping

## Abstract

**Purpose:**

To develop a mapping model to estimate EQ-5D-3L from the Knee Injury and Osteoarthritis Outcome Score (KOOS).

**Methods:**

The responses to EQ-5D-3L and KOOS questionnaires (*n* = 40,459 observations) were obtained from the Swedish National anterior cruciate ligament (ACL) Register for patients ≥ 18 years with the knee ACL injury. We used linear regression (LR) and beta-mixture (BM) for direct mapping and the generalized ordered probit model for response mapping (RM). We compared the distribution of the original data to the distributions of the data generated using the estimated models.

**Results:**

Models with individual KOOS subscales performed better than those with the average of KOOS subscale scores (KOOS_5_, KOOS_4_). LR had the poorest performance overall and across the range of disease severity particularly at the extremes of the distribution of severity. Compared with the RM, the BM performed better across the entire range of disease severity except the most severe range (KOOS_5_ < 25). Moving from the most to the least disease severity was associated with 0.785 gain in the observed EQ-5D-3L. The corresponding value was 0.743, 0.772 and 0.782 for LR, BM and RM, respectively. LR generated simulated EQ-5D-3L values outside the feasible range. The distribution of simulated data generated from the BM model was almost identical to the original data.

**Conclusions:**

We developed mapping models to estimate EQ-5D-3L from KOOS facilitating application of KOOS in cost-utility analyses. The BM showed superior performance for estimating EQ-5D-3L from KOOS. Further validation of the estimated models in different independent samples is warranted.

**Electronic supplementary material:**

The online version of this article (10.1007/s11136-019-02303-9) contains supplementary material, which is available to authorized users.

## Introduction

With the growing emphasis on the patients’ involvement in clinical decision-making, patient-reported outcome measures (PROMs) are increasingly used in the clinical settings to assess the effects of diseases and their treatments from the patient perspective [1]. In addition, generic preference-based PROMs, such as EQ-5D, have an important role in the assessment of health-related quality of life (HRQoL) and in calculating quality-adjusted life years (QALYs) for use in health economic evaluations [2]. QALYs combine HRQoL and survival into a single metric and is a common outcome measure applied in cost-utility analyses. However, clinical studies mostly use condition-specific PROMs which cannot be used to estimate QALYs [3]. In these situations, it is common to use statistical techniques (known as “mapping” or “cross walking”) to convert the responses on a condition-specific PROM to a generic preference-based PROM using datasets of patients that have responded to both measures simultaneously [2, 3]. While mapping studies are criticized for underestimating uncertainty and overprediction of poor health states, these are to some extent a sign of an inappropriate mapping model or inappropriate use and not a feature inherent in mapping [3]. While data on preference-based PROMs are preferable, mapping is a viable alternative when these data are not available [4].

The Knee Injury and Osteoarthritis Outcome Score (KOOS) is a commonly used knee-specific PROM intended for use in people across the lifespan with knee injury including anterior cruciate ligament (ACL) injury that can result in post-traumatic osteoarthritis [5]. The KOOS contains 42 items covering five subscales: pain, other symptoms, function in daily living (ADL), Function in sport and recreation (Sport/Rec) and knee-related quality of life (QoL) [5]. All items have five possible answer options ranged from 0 (no problems) to 4 (extreme problems). A normalized score (100 indicating no symptoms and 0 indicating extreme symptoms) is calculated for each subscale. To our best knowledge, there is no mapping model to estimate EQ-5D-3L values from the KOOS. To address this knowledge gap, we aimed to develop a mapping model to derive EQ-5D-3L values from the KOOS for use in cost-utility analyses among adult patients with ACL injury.

## Methods and materials

### Data

We used the data from the Swedish National ACL Register (www.aclregister.nu). This register was initiated in January 2005 comprising patients undergoing ACL reconstruction and ACL revision [6, 7]. The register coverage is estimated to exceed 90% of all surgical ACL procedures performed annually in Sweden [6]. The register uses a web-based protocol and the patients respond to the Swedish version of both EQ-5D-3L and KOOS before the ACL surgery and at 1, 2, 5 and 10 years after the operation. About 70% of patients respond to PROMs prior to operation and this number declines to 50% and 40% at 2 and 5 years follow up, respectively. (https://aclregister.nu/info/rapport2016en.pdf).

### Patients

We obtained the data on 52,584 observations for 25,169 patients operated between January 2005 and December 2014 from the Swedish ACL register. After exclusion of 12,125 observations (3678 missing responses to EQ-5D-3L, 3236 missing responses to the KOOS, 42 missing responses to both questionnaires and 5169 younger than 18 years when responded to the PROMs), a total of 40,459 observations (12,582 pre-operation, and 27,877 post-operation) from 21,854 patients were used for the analysis. We excluded those younger than 18 years since the UK utility weights were obtained from the adult population which may not reflect the preferences of children and adolescents and also dimensions of health relevant to children and adolescents may be different from adults [8].

### Statistical analysis

The conceptual overlap between the two measures used in mapping is important for acceptable performance of mapping algorithms [2]. Previous studies reported sufficient overlap between EQ-5D-3L and KOOS [9, 10]. The only dimension of the EQ-5D-3L that is not covered directly by the KOOS is anxiety/depression. We assessed the degree of overlap between the two instruments by calculating Spearman’s rank correlation coefficients between EQ-5D-3L index score and five KOOS subscales scores.

While linear regression is by far the most commonly used method to develop mapping models [4], it fails to account for some well-known characteristics of the EQ-5D-3L distribution such as the right and left bounding, a mass of observations at full health, a large gap between full health and the next feasible EQ-5D-3L value (e.g. no value between 1 and 0.883 in the UK value set) and multimodality of the distribution [11]. Therefore, response mapping and mixture models have gained popularity in developing mapping models [4]. In the current study, we used response mapping and mixture model in addition to linear regression.

For the response mapping, we used the generalized ordered probit model. The standard ordered models (probits or logits) assume the same coefficients for the explanatory variables across the different categories of dependent variable (parallel line assumption) and this has led to multinomial logit models being commonly used for the response mapping [12]. However, these models ignore the ordered nature of EQ-5D-3L data. The generalized ordered probit model relaxes the parallel line (proportional odds) assumption while accounting for the ordered nature of the EQ-5D-3L responses [12]. This allows the effects of the explanatory variables to vary with the point at which the categories of the dependent variable are dichotomized. In this study, we relaxed parallel line assumption for all explanatory variables. A separate model was estimated for each of five EQ-5D-3L dimensions and the probability of being at each of three levels (“no problems”, “some problems” and “extreme problems”) was calculated. Then based on these probabilities and the UK EQ-5D-3L tariff, the expected EQ-5D-3L value was computed mathematically [12, 13].

There has been an increasing popularity in the use of mixture models for mapping in recent years mainly due to their flexibility and the ability to capture multimodality of EQ-5D-3L data. The main concept in mixture modelling is that an underlying observed distribution can be represented by a mixture of distinct simpler distributions (components) with potential heterogeneity of covariates and their effects for each of these components [14]. The probability of being in each component is estimated using a multinomial logit model. In this study, we used a beta-mixture model which has recently been introduced by Gray et al. [15, 16] based on the truncated inflated beta regression model [17]. This is a two-part model including a multinomial logit model and a beta-mixture model. The multinomial logit model deals with the data at the boundaries and a mass of observations at full health and the mixture of beta distributions capture multimodality of the EQ-5D-3L data [15].

The KOOS was included in three alternative forms: individual KOOS subscales scores, the KOOS_5_ score (the average of the five KOOS subscales scores, ranged from 0 to 100 in our sample) and the KOOS_4_ score (the average of the KOOS subscales scores excluding the ADL subscale, as previously used in ACL injured populations [18], ranged from 1.25 to 100 in our sample). We also used the KOOS individual items but it caused convergence problem in beta-mixture model and we decided to not include them in our final analysis to ensure the models were comparable. For each of these alternatives, we applied a series of model specifications based on main terms, and main terms plus squared and square root terms (likelihood ratio test was used for exclusion of squared and square root terms). The models estimated for linear regression and response mapping are presented in Supplementary Tables 1 and 2. For beta-mixture model, we estimated different specifications with different numbers of components (starting with a one-component model equivalent to a beta regression model), with and without inclusion of the gap between full health and the next feasible value (UK EQ-5D-3L = 0.883), and with and without probability masses at full health and truncation point of the EQ-5D-3L distribution. An example of models estimated for a single specification is presented in Supplementary Table 3.

### Assessment of model performance

We used the Bayesian information criterion (BIC) to assess the goodness of fit of these specifications within each class of models, where a smaller BIC indicates a better model fit. The predictive ability of models was assessed using mean error (ME), mean absolute error (MAE) and root mean squared error (RMSE). The MAE is the mean of absolute differences between the observed and predicted EQ-5D-3L index scores, whilst the RMSE is defined as the squared root of the mean of squared differences between the observed and predicted EQ-5D-3L index scores. For each alternative form of KOOS (individual KOOS subscales scores, the KOOS_5_ and the KOOS_4_ scores) and each class of models (linear, response mapping, beta-mixture), we selected one model with the smallest BIC, and the lowest ME, MAE and RMSE in the whole sample and across the distribution of disease severity measured by the KOOS_5_/KOOS_4_ scores as *preferred model* (Supplementary Tables 4–12). Then, we selected one model as the *optimal model* for each class of models. In our decision to select the preferred models, we gave higher priority to the models with smallest BIC, while in selecting the optimal models higher priority was given to models with better predictive ability (in our study optimal models had both lower BIC and better predictive ability compared to other models).

An important application of mapping models is estimating EQ-5D-3L values in individual simulation-based cost-effectiveness models where many hypothetical individual patients with varying characteristics are simulated over a long time period or in trial based economic evaluations [11]. As a further assessment of model performance, we simulated data using the estimated models as the data generating process based on 100 replications for each observation in the sample (a total of 4,045,900 simulated EQ-5D data points) [12, 13]. The distribution of these simulated data was compared with the distribution of the observed EQ-5D-3L data. A model that correctly fits the EQ-5D-3L data should produce a distribution that resembles the distribution of the actual EQ-5D-3L data [2, 12, 13]. All analyses were performed in STATA v.15. We used the “goprobit” command [19] for response mapping and the “betamix” command [15] for beta-mixture model. Standard errors were adjusted for repeated observations from individual patients (using the “cluster” option). We used the “predict” post-estimation command for obtaining predicted values for linear and beta-mixture models. We did not transform the predictions outside the possible EQ-5D-3L range.

## Results

**Fig. 1:**
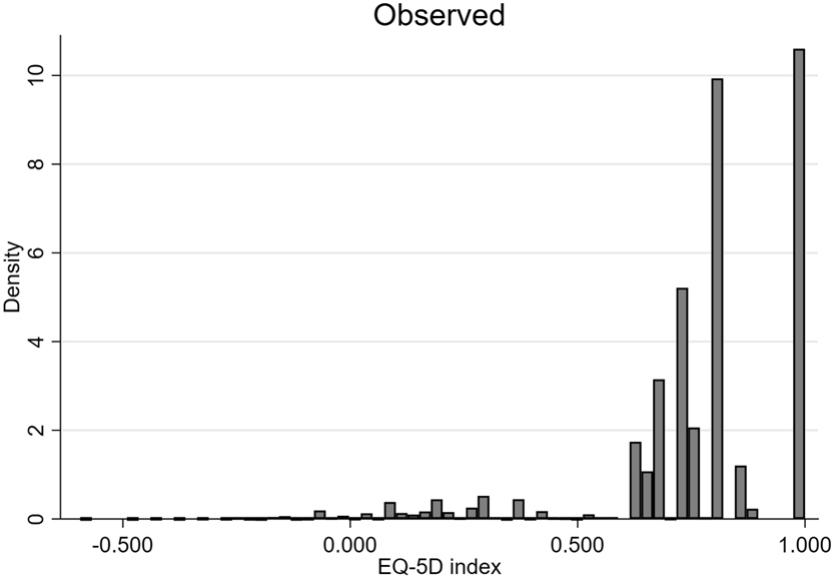
The distribution of EQ-5D-3L data in the sample

The patient sample had a mean (standard deviation) age of 29.1 (10.0) years and 42.3% were women at the date of ACL operation. The proportion of responses with some/extreme problems on EQ-5D-3L dimensions ranged from 1.6% in self-care to 68.2% in pain (Table 1). Across KOOS subscales the worst and best scores were reported for KOOS-QoL and KOOS-ADL, respectively. A total of 145 out of 243 possible EQ-5D-3L health states were observed. The full health (health state “11111”) was the most frequent health state (27.3%, Fig. 1) followed by health states “11121” (25.1%) and “11122” (11.0%). The Spearman rank correlation between EQ-5D-3L values and KOOS subscales ranged from 0.45 (Symptoms) to 0.56 (ADL) for pre-operation and from 0.66 (Symptoms) to 0.75 (QoL) for post-operation observations.

**Table 1:** Characteristics of the study sample, stratified by sex

	Pre-operation	Post-operation	Total
Number of patients	12,582	16,983	21,854
Number of observations	12,582	27,877	40,459
Mean (SD) age at operation, years	30.0 (9.4)	29.5 (10.3)	29.1 (10.0)
EQ-5D mobility			
No problems (%)	66.8	88.5	81.7
Some problems (%)	32.8	11.5	18.1
Extreme problems (%)	0.4	0.0	0.2
EQ-5D self-care			
No problems (%)	97.0	99.0	98.4
Some problems (%)	2.4	0.7	1.2
Extreme problems (%)	0.6	0.3	0.4
EQ-5D usual activities			
No problems (%)	54.1	82.5	73.6
Some problems (%)	35.9	16.6	22.6
Extreme problems (%)	10.0	0.9	3.8
EQ-5D pain			
No problems (%)	15.5	39.2	31.9
Some problems (%)	79.1	58.1	64.6
Extreme problems (%)	5.4	2.7	3.5
EQ-5D anxiety/depression			
No problems (%)	50.6	71.8	65.2
Some problems (%)	43.9	25.8	31.4
Extreme problems (%)	5.5	2.4	3.4
Proportion in full health (EQ-5D-3L = 1), %	9.0	35.5	27.3
Proportion reporting EQ-5D-3L < 0	1.8	0.9	1.2
Mean (SD) EQ-5D-3L index	0.66 (0.24)	0.81 (0.20)	0.77 (0.22)
Mean (SD) KOOS_5_	58.9 (16.5)	75.7 (17.8)	70.5 (19.1)
Mean (SD) KOOS_4_	53.1 (16.9)	71.9 (19.5)	66.1 (20.6)
Mean (SD) KOOS-pain	73.4 (17.8)	84.6 (15.9)	81.1 (17.3)
Mean (SD) KOOS-symptoms	68.7 (18.3)	77.9 (18.1)	75.1 (18.7)
Mean (SD) KOOS-activity of daily living	82.1 (17.6)	91.1 (13.5)	88.3 (15.5)
Mean (SD) KOOS-sports/recreation	38.3 (26.7)	64.5 (27.7)	56.3 (30.0)
Mean (SD) KOOS-quality of life	32.0 (17.3)	60.6 (24.0)	51.7 (25.8)

Proportions/means are reported across observations not patients

The estimates and full variance–covariance matrix of the preferred models based on individual subscales, KOOS_5_ and KOOS_4_ for three classes of models (linear regression, response mapping and beta-mixture) are reported in Supplementary Tables 4–12. In all three classes of models, the optimal models were those based on individual KOOS subscales.

In the optimal linear regression model (Supplementary Table 6), improvement in KOOS-Pain, Symptoms, ADL and QoL subscales scores (indicating better function) were associated with increase in EQ-5D-3L index score, even though for Symptoms the increase was at a lesser rate. The EQ-5D-3L improved up to a KOOS Sport/Rec score of 15, then it declined up to 50, and improved again once KOOS Sport/Rec exceeded 50. Older age and being female were associated with better EQ-5D-3L scores.

In the generalized ordered probit model, the inclusion of specific explanatory variables for each EQ-5D-3L dimension showed a better performance than the inclusion of the same explanatory variables for all EQ-5D-3L dimensions. The interpretation of the coefficients from the generalized ordered probit model is not straightforward and the inclusion of the squared and square root terms complicates this even further. In the optimal generalized ordered probit model (Supplementary Table 9), for the EQ-5D-3L mobility, self-care and usual activities, being female increased the probability of being at level 1 (“no problems”) and decreased the probability of being at level 3 (“severe problems”), all else equal. The opposite was true for the EQ-5D-3L pain dimension.

Our optimal beta-mixture model (Supplementary Table 12) was a three-component model including the gap between full health and the next feasible EQ-5D-3L value with a probability mass at full health (convergence was a problem with a four-component model). The three components centred on EQ-5D-3L values of 0.75, 0.29 and 0.71 with component membership probability of 0.88, 0.09 and 0.03, respectively. Different explanatory variables were included in predicting the components mean, probability of component membership and probability of being in full health. The excel calculator in Supplement can be used to estimate EQ-5D-3L values using the optimal linear, beta-mixture and generalized ordered probit models.

In the full sample, while the linear regression provided the closest estimate to the observed mean (including a constant in a linear regression ensures this is the case), it had larger MAE and RMSE than the response mapping and mixture models (Table 2). In addition, across the range of disease severity measured by the KOOS_5_ scores, the response mapping and mixture model outperformed linear regression in terms of all summary measures and importantly this was more profound (the highest proportional improvements in the MAE and RMSE) at the extremes of the distribution of disease severity. Compared with the response mapping model, the beta-mixture model estimated closer mean to the observed mean in overall and across the range of the KOOS_5_ score except those < 25 (most severe). For all models the magnitude of MAE and RMSE rose with the severity of the disease. The results were generally similar when we measured disease severity by the KOOS_4_ scores (Table 3).

**Table 2:** Prediction performance of optimal models in full sample and across the range of disease severity (measured by KOOS_5_ scores)

Sample and summary statistics	Linear regression^a^	Response mapping^b^	Beta-mixture model^c^
Full sample (*n* = 40,459)			
ME	**1.65 × 10**^**−17**^	0.0026	− 0.0003
MAE	0.1037	0.0996	**0.0988**
RMSE	0.1505	**0.1489**	0.1490
KOOS_5_ 0 to < 25 (*n* = 543)			
ME	− 0.0359	− **0.0005**	**− **0.0145
MAE	0.2305	**0.2140**	0.2177
RMSE	0.2776	**0.2671**	0.2691
KOOS_5_ 25 to < 50 (*n* = 6069)			
ME	0.0067	0.0078	**− 0.0001**
MAE	0.1948	0.1897	**0.1877**
RMSE	0.2358	0.2340	**0.2340**
KOOS_5_ 50 to < 70 (*n* = 11,370)			
ME	0.0030	0.0007	**0.0004**
MAE	0.0951	0.0923	**0.0922**
RMSE	0.1504	**0.1493**	0.1495
KOOS_5_ 70 to < 85 (*n* = 11,535)			
ME	− 0.0107	0.0012	**0.0007**
MAE	0.0874	0.0831	**0.0830**
RMSE	0.1242	0.1229	**0.1229**
KOOS_5_ 85 to ≤ 100 (*n* = 10,942)			
ME	0.0062	0.0032	**− 0.0015**
MAE	0.0729	0.0693	**0.0671**
RMSE	0.0962	0.0947	**0.0946**

The closest fit to the observed data in bold

*ME* mean error (observed minus predicted), *MAE* mean absolute error, *RMSE* root mean squared error

^a^A model specification with KOOS subscales, age, sex, squared and square root terms for KOOS-Pain and Sport, squared term for KOOS-ADL and square root term for KOOS-QoL. Model estimates are presented in Supplementary Table 6

^b^Model estimates are presented in Supplementary Table 9

^c^A three-component model including the gap between full health and the next feasible EQ-5D-3L value with a probability mass at full health. Model estimates are presented in Supplementary Table 12

**Table 3:** Prediction performance of optimal models across the range of disease severity (measured by KOOS_4_ scores)

Sample and summary statistics	Linear regression^a^	Response mapping^b^	Beta-mixture model^c^
KOOS_4_ 0 to < 25 (*n* = 1136)			
ME	− 0.0246	0.0121	− **0.0017**
MAE	0.2472	**0.2364**	0.2390
RMSE	0.2821	**0.2760**	0.2768
KOOS_4_ 25 to < 50 (*n* = 8528)			
ME	0.0088	0.0035	− **0.0017**
MAE	0.1633	0.1582	**0.1565**
RMSE	0.2114	0.2098	**0.2098**
KOOS_4_ 50 to < 70 (*n* = 11,892)			
ME	− 0.0025	**0.0011**	0.0012
MAE	0.0826	**0.0806**	0.0806
RMSE	0.1363	**0.1354**	0.1355
KOOS_4_ 70 to < 85 (*n* = 10,167)			
ME	− 0.0084	0.0011	− **0.0002**
MAE	0.0957	0.0912	**0.0910**
RMSE	0.1231	0.1218	**0.1218**
KOOS_4_ 85 to ≤ 100 (*n* = 8736)			
ME	0.0078	0.0040	− **0.0009**
MAE	0.0647	0.0607	**0.0580**
RMSE	0.0889	0.0870	**0.0870**

The closest fit to the observed data in bold

*ME* mean error (observed minus predicted), *MAE* mean absolute error, *RMSE* root mean squared error

^a^A model specification with KOOS subscales, age, sex, squared and square root terms for KOOS-Pain and Sport, squared term for KOOS-ADL and square root term for KOOS-QoL. Model estimates are presented in Supplementary Table 6

^b^Model estimates are presented in Supplementary Table 9

^c^A three-component model including the gap between full health and the next feasible EQ-5D-3L value with a probability mass at full health. Model estimates are presented in Supplementary Table 12

Moving from the lowest (< 25) to the highest (≥ 85) level of KOOS_5_ score was associated with 0.785 change in EQ-5D-3L values in the observed data. The corresponding value was 0.743, 0.782 and 0.772 for linear, response mapping and beta-mixture model, respectively, indicating a difference of 0.038 between models.

**Fig. 2:**
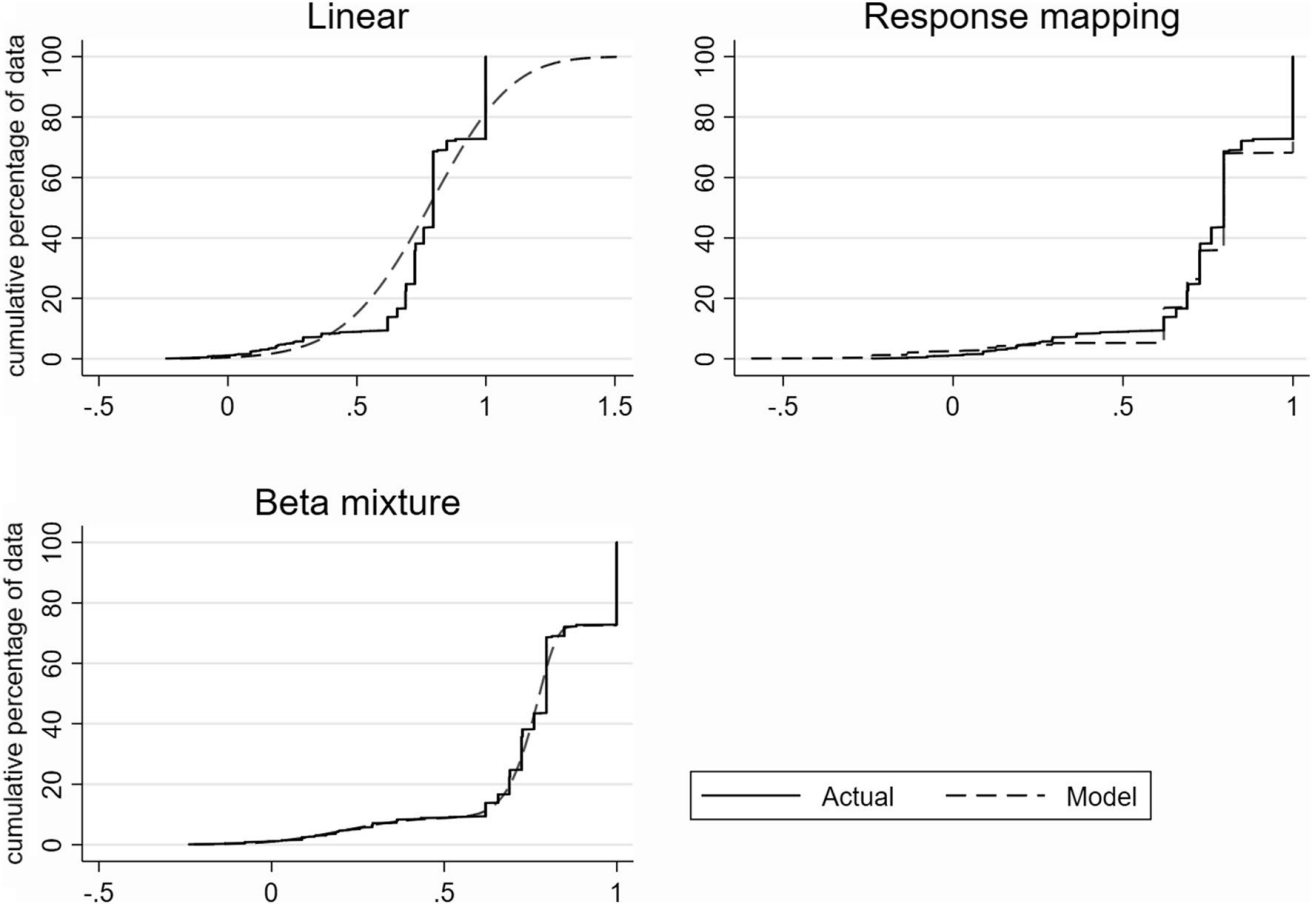
The cumulative percentage of observed EQ-5D-3L data vs. simulated data from the best performed models

The distribution of simulated data showed that linear regression clearly failed to account for main characteristics of the original data (Table 4; Fig. 2). While linear regression generated EQ-5D-3L values that fall way outside the feasible range (− 0.594 to 1.0), neither the response mapping nor the beta-mixture model suffer from this limitation by design. In contrast to the beta-mixture model, the response mapping take into account the discrete nature of the EQ-5D-3L data. The data generated by the beta-mixture model more closely resemble the original data.

**Table 4:** Summary statistics of the observed and simulated data sets generated using the optimal models

	Observed	Linear regression^a^	Response mapping^b^	Beta-mixture model^c^
Mean	0.766	**0.767**	0.781	0.767
Variance	0.049	0.069	0.055	**0.050**
Skewness	− 1.633	− 0.268	− 2.289	− **1.644**
Kurtosis	6.478	3.289	10.459	**6.571**
Minimum	− 0.594	− 1.112	− **0.594**	− **0.594**
Maximum	1.000	2.002	**1.000**	**1.000**
EQ-5D-3L = 1, %	27.26	0.0	31.81	**27.26**
EQ-5D-EL > 1, %	0.0	18.59	**0.0**	**0.0**
EQ-5D-EL < 0, %	1.16	0.47	2.52	**1.01**
Percentiles				
1%	− 0.016	0.097	− 0.239	− **0.002**
5%	0.228	0.320	0.293	**0.223**
10%	0.620	0.429	**0.620**	0.592
25%	0.725	0.599	0.689	**0.711**
50%	0.796	0.777	**0.796**	0.777
75%	1.000	0.946	**1.000**	**1.000**
90%	1.000	1.093	**1.000**	**1.000**
95%	1.000	1.179	**1.000**	**1.000**
99%	1.000	1.337	**1.000**	**1.000**

The closest fit to the observed data in bold

^a^A model specification with KOOS subscales, age, sex, squared and square root terms for KOOS-Pain and Sport, squared term for KOOS-ADL and square root term for KOOS-QoL. Model estimates are presented in Supplementary Table 6

^b^Model estimates are presented in Supplementary Table 9

^c^A three-component model including the gap between full health and the next feasible EQ-5D-3L value with a probability mass at full health. Model estimates are presented in Supplementary Table 12

## Discussion

This is, to our knowledge, the first study to develop mapping models to predict EQ-5D-3L values from the KOOS. This facilitates the application of KOOS in cost-utility analyses in ACL studies when the directly collected EQ-5D-3L data are not available. The overall MAE (0.099 to 0.104) and RMSE (0.149 to 0.151) found in our study were comparable to those generally reported in the mapping literature (from 0.0011 to 0.19 for MAE, and from 0.084 to 0.20 for RMSE) [3]. Our results also confirmed that linear regression might not be appropriate for mapping. The three-component beta-mixture model fit the data generally better and generated simulated data that more closely resembled the observed data.

Our results showed that regardless of econometric technique, the models based on the individual KOOS subscales had better performance than those based on the average of the subscale scores (i.e. KOOS_5_ and KOOS_4_). However, individual scores are not always available to map from and models using average scores are needed. It should be noted that previous studies suggested that the KOOS-ADL subscale might have poor content validity for young adults with ACL injury [20], however its inclusion in our study improved the predictive ability of our mapping models.

In addition to linear regression, we applied two other statistical techniques based on recent advances in modelling EQ-5D data: beta-mixture model, and generalized ordered probit model. Both these techniques outperformed linear regression overall and across the range of disease severity particularly at the extremes of the distribution of disease severity. This is in agreement with recent evidence suggesting that the characteristics of EQ-5D-3L data make linear regression inappropriate for mapping [11, 12, 21]. A recent systematic review found that the proportion of mapping studies using solely linear regression declined from 49% in 1997–2011 to 13% in 2014–2016 [4]. Some studies reported small differences in predictive ability of linear compared to other models [22–24] including the response mapping and mixture models [14]. However, these studies solely relied on the observed mean EQ-5D-3L value and dismiss the data generating process of these models and its importance for simulation-based cost-effectiveness analyses. Furthermore, while due to regress toward mean, linear regression might have better performance in overall, it generally has poor performance compared with other models at the extreme of disease severity [21]. Moreover, it is important to bear in mind that due to very limited range of EQ-5D-3L data, small differences in prediction errors should not be overlooked [25].

For mixture modelling, we used beta-mixture model which, to our knowledge, has been applied only in one previous mapping study where it marginally outperformed the adjusted limited dependent variable mixture model [16]. Our preferred beta-mixture model was a three-component model including the gap between full health and the next feasible EQ-5D-3L value with a probability mass at full health. While adding more components to beta-mixture model resulted in convergence problem in our study, assessing the performance of models with larger number of components in other data sets is a subject for future research. We have also estimated models with the probability masses at both full health and truncation point, but these had poorer fit compared to our preferred model. This was not unexpected because only 0.6% of the observations were at the EQ-5D-3L truncation point (0.883). It also should be noted that our beta-mixture model was estimated using the UK value set reported by Dolan et al. [26]. Different countries have different value sets and the mapping function given here is not necessarily applicable to all other countries. The response mapping estimates reported here could be used for alternative countries by attaching the corresponding value set in the second step if a better performing mapping function is not available under the additional assumption that the responses to the questionnaire would be similar across different countries.

To our knowledge, the predictive ability of beta-mixture model and response mapping has not been previously compared. Our results demonstrated that while both techniques are appealing for the purposes of mapping, the beta-mixture model performed better across the entire range of disease severity except most severe range in this dataset. However, only 1.3% of observations were at this extreme level of disease severity. In line with this, two previous mapping studies reported that while a limited dependent variable mixture model outperformed the response mapping, this was not universal across entire range of disease severity [12, 25]. Furthermore, the simulated data produced from our preferred beta-mixture model had very close summary statistics to those in the original data. It should be noted that our models were developed in a sample of young patients with ACL injury and hence their application in other populations (e.g. older age groups, patients with other knee problems) should be taken with caution [27].

Developing the first models for mapping EQ-5D-3L from the KOOS, using a large data set covering a wide range of disease severity, and the first comparison of beta-mixture model and response mapping are the main strengths of the current study. However, several limitations of the study should be acknowledged. First, a very small portion of the observations were at the most severe range (< 25) for the KOOS-Pain (0.5%), Symptoms (0.7%) and ADL (0.3%) subscales which might influence the generalizability of our models to data sets with a greater portion of patients at this severe range. However, it should be noted that these subscales are generally less affected compared with Sport/Rec and QoL subscales in patients with ACL injury [28, 29]. For example, in the Multicenter Orthopaedic Outcomes Network cohort [28], the 25th percentile for Pain, Symptoms and ADL subscales were 64, 57 and 74 which are comparable to our data. Second, we were not able to validate our models on an external data set. While some mapping studies randomly split their data into an “estimation” and a “validation” subsamples, this approach is not universally recommended [2, 25]. Mapping models are inputs into subsequent analyses and, therefore, validation should take this second step into account. A mapping model could be used to, either predict a conditional mean, or simulate individual level data from the conditional distribution. We present measures in the paper on which to judge the internal validity of models in those two areas. External validity will be dependent on subsequent analyses and cannot be generalized. Third, we used the data from the Swedish ACL register and the high percentage of nonresponses to the PROMs, particularly in follow up, is of concern. Fourth, measurement error in the predictors is a potential problem in mapping models and remains an area of future research.

## Conclusions

To facilitate the use of KOOS in cost-utility analyses, we developed the first set of models to estimate the EQ-5D-3L values from the KOOS using data from adult patients with ACL injury. Our results confirmed inadequacy of linear regression for mapping and also showed that beta-mixture model had superior performance compared with response mapping. Further research is warranted to investigate predictive ability of the estimated models in other data sets or other settings, e.g. different age distribution and other knee conditions.

## Electronic supplementary material

Below is the link to the electronic supplementary material.

Supplementary material 1 (DOCX 13 kb)

Supplementary material 2 (DOCX 19 kb)

Supplementary material 3 (DOCX 13 kb)

Supplementary material 4 (XLS 32 kb)

Supplementary material 5 (XLS 27 kb)

Supplementary material 6 (XLS 32 kb)

Supplementary material 7 (XLS 55 kb)

Supplementary material 8 (XLS 58 kb)

Supplementary material 9 (XLS 119 kb)

Supplementary material 10 (XLS 57 kb)

Supplementary material 11 (XLS 57 kb)

Supplementary material 12 (XLS 164 kb)

Supplementary material 13 (XLSX 7281 kb)
