# Supplementary material for: Mapping EQ-5D-3L from the Knee Injury and Osteoarthritis Outcome Score (KOOS)
Source: Qual Life Res. 2019 Sep 20;29(1):265–74. doi: 10.1007/s11136-019-02303-9 (PMC6962127; doi:10.1007/s11136-019-02303-9)
Supplement: Supplementary file 1 — Supplementary material 1 (DOCX 13 kb) [file 11136_2019_2303_MOESM1_ESM.docx]

Table 1. The model specifications estimated using linear regression.

| Model # | Covariates | BIC | ME | MAE | RMSE |
| --- | --- | --- | --- | --- | --- |
| 1 | KOOS_4_ score, age, sex | -34556.6 | 1.04e-16 | 0.1102 | 0.1578 |
| 2 | KOOS_4_ score, age, sex, squared and square root (KOOS_4_ score, age) | -36278.1 | -2.63e-16 | 0.1058 | 0.1544 |
| 3 | KOOS_4_ score, age, sex, squared and square root KOOS_4_ score, squared age | -36287.7 | -4.97e-16 | 0.1058 | 0.1544 |
| 4 | KOOS_4_ score, age, sex, squared and square root KOOS_4_ score, square root age | -36286.6 | -4.34e-17 | 0.1058 | 0.1544 |
| 5 ^a^ | KOOS_4_ score, age, sex, squared and square root KOOS_4_ score | -36291.9 | -1.49e-16 | 0.1058 | 0.1544 |
| 6 | KOOS_5_ score, age, sex | -35517.7 | -1.09e-16 | 0.1093 | 0.1559 |
| 7 | KOOS_5_ score, age, sex, squared and square root (KOOS_5_ score, age) | -36682.3 | 5.81e-16 | 0.1057 | 0.1536 |
| 8 | KOOS_5_ score, age, sex, squared and square root KOOS_5_ score, squared age | -36691.5 | 1.26e-15 | 0.1057 | 0.1536 |
| 9 | KOOS_5_ score, age, sex, squared and square root KOOS_5_ score, square root age | -36690.7 | 4.45e-16 | 0.1057 | 0.1536 |
| 10 ^a^ | KOOS_5_ score, age, sex, squared and square root KOOS_5_ score | -36699.0 | 9.63e-16 | 0.1057 | 0.1536 |
| 11 | Pain, Symptoms, ADL, Sport/Rec, QoL, age , sex | -37024.5 | 1.47e-16 | 0.1067 | 0.1530 |
| 12 | Pain, Symptoms, ADL, Sport/Rec, QoL, age , sex, squared and square root (Pain, Symptoms, ADL, Sport/Rec, QoL, age) | -38262.1 | -2.02e-16 | 0.1035 | 0.1504 |
| 13 | Pain, ADL, Sport/Rec, QoL, age , sex, squared and square root (Pain, Symptoms, ADL, Sport/Rec, age), square root QoL | -38272.7 | -1.14e-19 | 0.1035 | 0.1504 |
| 14 | Pain, ADL, Sport/Rec, QoL, age , sex, squared and square root (Pain, ADL, Sport/Rec, age), squared Symptoms, square root QoL | -38280.3 | -3.00e-16 | 0.1035 | 0.1504 |
| 15 | Pain, ADL, Sport/Rec, QoL, age , sex, squared and square root (Pain, ADL, Sport/Rec, age), square root QoL | -38289.6 | -3.27e-16 | 0.1036 | 0.1504 |
| 16 | Pain, ADL, Sport/Rec, QoL, age , sex, squared and square root (Pain, ADL, Sport/Rec), square root QoL, squared age | -38297.3 | -2.59e-16 | 0.1036 | 0.1504 |
| 17 | Pain, ADL, Sport/Rec, QoL, age , sex, squared and square root (Pain, Sport/Rec), square root QoL, squared (age, ADL) | -38296.7 | -3.49e-17 | 0.1036 | 0.1504 |
| 18 ^a, b^ | Pain, ADL, Sport/Rec, QoL, age , sex, squared and square root (Pain, Sport/Rec), square root QoL, squared ADL | -38300.8 | -3.21e-16 | 0.1036 | 0.1504 |

^a^ The preferred model for each form of KOOS alternative.

^b^ The optimal model in linear class.
