# Supplementary material for: Mapping EQ-5D-3L from the Knee Injury and Osteoarthritis Outcome Score (KOOS)
Source: Qual Life Res. 2019 Sep 20;29(1):265–74. doi: 10.1007/s11136-019-02303-9 (PMC6962127; doi:10.1007/s11136-019-02303-9)
Supplement: Supplementary file 2 — Supplementary material 2 (DOCX 19 kb) [file 11136_2019_2303_MOESM2_ESM.docx]

Table 2. The model specifications estimated using generalized ordered probit model.

| Model # | Mobility | | | Self-care | | | Usual activities | | | Pain | | | Anxiety | | | Overall | | | |
| --- | --- | --- | --- | --- | --- | --- | --- | --- | --- | --- | --- | --- | --- | --- | --- | --- | --- | --- | --- |
|  | Covariates | BIC | Covariates | | BIC | Covariates | | BIC | Covariates | | BIC | Covariates | | BIC | BIC | | ME | MAE | RMSE |
| 1 | KOOS_4_ score, age, sex | 23123.0 | Same as mobility | | 6401.3 | Same as mobility | | 41863.1 | Same as mobility | | 37917.2 | Same as mobility | | 49211.5 | 158516.1 | | 0.00306 | 0.1084 | 0.1544 |
| 2 | KOOS_4_ score, age, sex, squared and square root (KOOS_4_ score, age) | 23125.7 | Same as mobility | | 6344.6 | Same as mobility | | 41794.5 | Same as mobility | | 36845.2 | Same as mobility | | 49004.8 | 157114.8 | | 0.00315 | 0.1038 | 0.1536 |
| 3 | KOOS_4_ score, age, sex, squared and square root KOOS_4_ score, squared age | 23108.0 | Same as mobility | | 6329.9 | Same as mobility | | 41773.7 | Same as mobility | | 36829.8 | Same as mobility | | 48989.3 | 157030.6 | | 0.00315 | 0.1038 | 0.1536 |
| 4 | KOOS_4_ score, age, sex, squared and square root KOOS_4_ score, square root age | 23105.9 | Same as mobility | | 6329.0 | Same as mobility | | 41774.3 | Same as mobility | | 36827.2 | Same as mobility | | 48992.8 | 157029.3 | | 0.00315 | 0.1038 | 0.1536 |
| 5 | KOOS_4_ score, age, sex, squared KOOS_4_ score, square root age | 23088.0 | Same as mobility | | 6342.0 | Same as mobility | | 41794.1 | Same as mobility | | 36866.1 | Same as mobility | | 48988.0 | 157078.3 | | 0.00294 | 0.1046 | 0.1536 |
| 6 | KOOS_4_ score, age, sex, squared and square root KOOS_4_ score | 23099.1 | Same as mobility | | 6316.3 | Same as mobility | | 41763.4 | Same as mobility | | 36850.0 | Same as mobility | | 48988.7 | 157017.5 | | 0.00313 | 0.1038 | 0.1536 |
| 7 ^a^ | Model 5 | 23088.0 | Model 6 | | 6316.3 | Model 6 | | 41763.4 | Model 4 | | 36827.2 | Model 6 | | 48988.7 | 156983.6 | | 0.00314 | 0.1038 | 0.1536 |
| 8 | KOOS_5_ score, age, sex | 22485.5 | Same as mobility | | 6282.4 | Same as mobility | | 41842.0 | Same as mobility | | 37925.5 | Same as mobility | | 49310.2 | 157845.6 | | 0.00296 | 0.1076 | 0.1535 |
| 9 | KOOS_5_ score, age, sex, squared and square root (KOOS_5_ score, age) | 22528.3 | Same as mobility | | 6227.5 | Same as mobility | | 41673.2 | Same as mobility | | 36634.0 | Same as mobility | | 48989.3 | 156052.3 | | 0.00302 | 0.1032 | 0.1526 |

Table 11. Continue.

| Model # | Mobility | | | Self-care | | | Usual activities | | | Pain | | | Anxiety | | | Overall | | | |
| --- | --- | --- | --- | --- | --- | --- | --- | --- | --- | --- | --- | --- | --- | --- | --- | --- | --- | --- | --- |
|  | Covariates | BIC | Covariates | | BIC | Covariates | | BIC | Covariates | | BIC | Covariates | | BIC | BIC | | ME | MAE | RMSE |
| 10 | KOOS_5_ score, age, sex, squared and square root KOOS_5_ score, squared age | 22510.7 | Same as mobility | | 6213.0 | Same as mobility | | 41652.4 | Same as mobility | | 36618.7 | Same as mobility | | 48974.3 | 155969.0 | | 0.00301 | 0.1032 | 0.1526 |
| 11 | KOOS_5_ score, age, sex, squared and square root KOOS_5_ score, square root age | 22508.9 | Same as mobility | | 6212.2 | Same as mobility | | 41652.9 | Same as mobility | | 36616.1 | Same as mobility | | 48977.5 | 155967.6 | | 0.00301 | 0.1032 | 0.1526 |
| 12 | KOOS_5_ score, age, sex, squared KOOS_5_ score, square root age | 22498.9 | Same as mobility | | 6217.1 | Same as mobility | | 41688.8 | Same as mobility | | 36690.3 | Same as mobility | | 48980.4 | 156075.5 | | 0.00267 | 0.1041 | 0.1527 |
| 13 | KOOS_5_ score, age, sex, squared and square root KOOS_5_ score | 22497.7 | Same as mobility | | 6198.6 | Same as mobility | | 41639.1 | Same as mobility | | 36644.2 | Same as mobility | | 48970.7 | 155950.3 | | 0.00300 | 0.1033 | 0.1526 |
| 14 ^a^ | Model 8 | 22485.5 | Model 13 | | 6198.6 | Model 13 | | 41639.1 | Model 11 | | 36616.1 | Model 13 | | 48970.7 | 155910.0 | | 0.00300 | 0.1032 | 0.1526 |
| 15 | P, S, A, Q, SR, age, sex | 22249.0 | Same as mobility | | 6160.4 | Same as mobility | | 41069.4 | Same as mobility | | 36791.2 | Same as mobility | | 47627.4 | 153897.5 | | 0.00269 | 0.1039 | 0.1498 |
| 16 | P, S, A, Q, SR, age, sex, squared and square root (P, S, A, Q, SR, age) | 22312.1 | Same as mobility | | 6297.7 | Same as mobility | | 41082.6 | Same as mobility | | 35198.0 | Same as mobility | | 47520.6 | 152411.0 | | 0.00268 | 0.0997 | 0.1489 |
| 17 | P, S, A, Q, SR, age, sex, squared and square root SR, squared (A, Q), square root age | 22177.7 | Same as mobility | | 6183.9 | Same as mobility | | 40959.8 | Same as mobility | | 35619.9 | Same as mobility | | 47409.2 | 152350.5 | | 0.00256 | 0.1004 | 0.1491 |
| 18 | P, S, A, Q, SR, age, sex, squared and square root SR, squared (A, Q), squared age | 22179.0 | Same as mobility | | 6184.7 | Same as mobility | | 40958.9 | Same as mobility | | 35622.8 | Same as mobility | | 47404.2 | 152349.6 | | 0.00256 | 0.1004 | 0.1491 |

Table 11. Continue.

| Model # | Mobility | | | Self-care | | | Usual activities | | | Pain | | | Anxiety | | | Overall | | | |
| --- | --- | --- | --- | --- | --- | --- | --- | --- | --- | --- | --- | --- | --- | --- | --- | --- | --- | --- | --- |
|  | Covariates | BIC | Covariates | | BIC | Covariates | | BIC | Covariates | | BIC | Covariates | | BIC | BIC | | ME | MAE | RMSE |
| 19 | P, S, A, Q, SR, age, sex, squared and square root (A, age) | 22237.5 | Same as mobility | | 6152.3 | Same as mobility | | 41063.1 | Same as mobility | | 36323.8 | Same as mobility | | 47588.2 | 153364.9 | | 0.00257 | 0.1032 | 0.1494 |
| 20 | P, S, A, Q, SR, age, sex, squared and square root (P, SR), squared (S, A, Q, age) | 22235.4 | Same as mobility | | 6232.4 | Same as mobility | | 41003.1 | Same as mobility | | 35145.4 | Same as mobility | | 47444.8 | 152061.1 | | 0.00259 | 0.0997 | 0.1489 |
| 21 | P, S, A, Q, SR, age, sex, squared and square root (P, Q, SR), squared (S, A), square root age | 22251.6 | Same as mobility | | 6250.8 | Same as mobility | | 41023.4 | Same as mobility | | 35145.6 | Same as mobility | | 47470.7 | 152142.0 | | 0.00267 | 0.0997 | 0.1489 |
| 22 | P, S, A, Q, SR, age, sex, squared and square root (age), squared (S, A, Q, SR) | 22204.7 | Same as mobility | | 6195.2 | Same as mobility | | 40992.7 | Same as mobility | | 35552.4 | Same as mobility | | 47402.3 | 152347.3 | | 0.00257 | 0.1004 | 0.1492 |
| 23 | P, S, A, Q, SR, age, sex, squared and square root (SR), squared (A, Q) | 22164.0 | Same as mobility | | 6168.4 | Same as mobility | | 40949.3 | Same as mobility | | 35633.5 | Same as mobility | | 47409.1 | 152324.2 | | 0.00255 | 0.1004 | 0.1491 |
| 24 | P, S, A, Q, SR, age, sex, squared and square root (A, age), square root S | 22257.5 | Same as mobility | | 6169.4 | Same as mobility | | 41071.9 | Same as mobility | | 36225.9 | Same as mobility | | 47583.0 | 153307.6 | | 0.00250 | 0.1030 | 0.1494 |
| 25 | P, S, A, Q, SR, age, sex, squared and square root (P, SR), squared (A, Q, age) | 22214.9 | Same as mobility | | 6215.6 | Same as mobility | | 40988.1 | Same as mobility | | 35140.0 | Same as mobility | | 47437.3 | 151996.0 | | 0.00258 | 0.0998 | 0.1489 |

Table 11. Continue.

| Model # | Mobility | | Self-care | | Usual activities | | Pain | | Anxiety | | Overall | | | |
| --- | --- | --- | --- | --- | --- | --- | --- | --- | --- | --- | --- | --- | --- | --- |
|  | Covariates | BIC | Covariates | BIC | Covariates | BIC | Covariates | BIC | Covariates | BIC | BIC | ME | MAE | RMSE |
| 26 | P, S, A, Q, SR, age, sex, squared and square root (P, SR), squared (A, Q), square root (S, age) | 22233.9 | Same as mobility | 6231.2 | Same as mobility | 41004.9 | Same as mobility | 35147.9 | Same as mobility | 47452.9 | 152070.8 | 0.00258 | 0.0998 | 0.1489 |
| 27 | P, S, A, Q, SR, age, sex, squared and square root (P, SR), squared (A, Q), square root age | 22213.6 | Same as mobility | 6214.4 | Same as mobility | 40989.1 | Same as mobility | 35137.1 | Same as mobility | 47442.4 | 151996.6 | 0.00258 | 0.0998 | 0.1489 |
| 28 ^a, b^ | Model 23 | 22164.0 | Model 19 | 6152.3 | Model 23 | 40949.3 | Model 27 | 35137.1 | Model 22 | 47402.3 | 151805.0 | 0.00255 | 0.0996 | 0.1489 |

P: KOOS-Pain, S: KOOS-Symptoms, A: KOOS-ADL, SR: KOOS-Sport/Rec, Q: KOOS-QoL

^a^ The preferred model for each form of KOOS alternative.

^b^ The optimal model in response mapping class.
