# Supplementary material for: Mapping EQ-5D-3L from the Knee Injury and Osteoarthritis Outcome Score (KOOS)
Source: Qual Life Res. 2019 Sep 20;29(1):265–74. doi: 10.1007/s11136-019-02303-9 (PMC6962127; doi:10.1007/s11136-019-02303-9)
Supplement: Supplementary file 3 — Supplementary material 3 (DOCX 13 kb) [file 11136_2019_2303_MOESM3_ESM.docx]

Table 3. Beta-mixture models estimated for a single specification of covariates.

|  | Covariates of the mean | Covariates of probability mass | Covariates of component probabilities | # of components | Gap between full health and next feasible value | Position of probability masses |
| --- | --- | --- | --- | --- | --- | --- |
| 1 | KOOS_4_ score, age, sex | - | - | 1 | No | - |
| 2 | KOOS_4_ score, age, sex | KOOS_4_ score, age, sex | - | 1 | No | Full health |
| 3 | KOOS_4_ score, age, sex | KOOS_4_ score, age, sex | - | 1 | Yes | Full health |
| 4 | KOOS_4_ score, age, sex | KOOS_4_ score, age, sex | - | 1 | Yes | Full health, truncation point |
| 5 | KOOS_4_ score, age, sex | - | - | 2 | No | Full health |
| 6 | KOOS_4_ score, age, sex | KOOS_4_ score, age, sex | - | 2 | No | Full health |
| 7 | KOOS_4_ score, age, sex | KOOS_4_ score, age, sex | - | 2 | Yes | Full health |
| 8 | KOOS_4_ score, age, sex | KOOS_4_ score, age, sex | - | 2 | Yes | Full health, truncation point |
| 9 | KOOS_4_ score, age, sex | KOOS_4_ score, age, sex | KOOS_4_ score, age, sex | 2 | No | Full health |
| 10 | KOOS_4_ score, age, sex | KOOS_4_ score, age, sex | KOOS_4_ score, age, sex | 2 | Yes | Full health |
| 11 | KOOS_4_ score, age, sex | KOOS_4_ score, age, sex | KOOS_4_ score, age, sex | 2 | Yes | Full health, truncation point |
| 12 | KOOS_4_ score, age, sex | - | - | 3 | No | Full health |
| 13 | KOOS_4_ score, age, sex | KOOS_4_ score, age, sex | - | 3 | No | Full health |
| 14 | KOOS_4_ score, age, sex | KOOS_4_ score, age, sex | - | 3 | Yes | Full health |
| 15 | KOOS_4_ score, age, sex | KOOS_4_ score, age, sex | - | 3 | Yes | Full health, truncation point |
| 16 | KOOS_4_ score, age, sex | KOOS_4_ score, age, sex | KOOS_4_ score, age, sex | 3 | No | Full health |
| 17 | KOOS_4_ score, age, sex | KOOS_4_ score, age, sex | KOOS_4_ score, age, sex | 3 | Yes | Full health |
| 18 | KOOS_4_ score, age, sex | KOOS_4_ score, age, sex | KOOS_4_ score, age, sex | 3 | Yes | Full health, truncation point |
